# Systematic review of association between critical errors in inhalation and health outcomes in asthma and COPD

**DOI:** 10.1038/s41533-018-0110-x

**Published:** 2018-11-16

**Authors:** Janwillem W. H. Kocks, Henry Chrystyn, Job van der Palen, Mike Thomas, Louisa Yates, Sarah H. Landis, Maurice T. Driessen, Mugdha Gokhale, Raj Sharma, Mathieu Molimard

**Affiliations:** 1 General Practitioners Research Institute, Groningen, The Netherlands; 2 Inhalation Consultancy Ltd, Yeadon, UK; 3 Department of Research Methodology, Measurement, and Data Analysis, Faculty of Behavioural, Management and Social Sciences, and Department of Epidemiology, Medisch Spectrum Twente, University of Twente, Enschede, The Netherlands; 4 Primary Care and Population Sciences, University of Southampton, Southampton, UK; 5 NIHR Southampton Biomedical Research Centre, Southampton, UK; 6 NIHR Wessex Collaboration for Leadership in Applied Health Research and Care (CLAHRC), Southampton, UK; 7 GSK, Value Evidence & Outcomes, Brentford, UK; 8 GSK, Real World Evidence & Epidemiology, Uxbridge, UK; 9 GSK, Real World Evidence & Epidemiology, Collegeville, PA USA; 10 Department of Pharmacology, University of Bordeaux, Bordeaux, France

## Abstract

Inhaled medications are the cornerstone of treatment and management of asthma and COPD. However, inhaler device errors are common among patients and have been linked with reduced symptom control, an increased risk of exacerbations, and increased healthcare utilisation. These observations have prompted GINA (Global INitiative for Asthma) and GOLD (Global initiative for chronic Obstructive Lung Disease) to recommend regular assessment of inhaler technique in a bid to improve therapeutic outcomes. To better define the relationship between device errors and health outcomes (clinical outcomes, quality of life, and healthcare utilisation) in asthma and COPD, we conducted a systematic review of the literature, with a particular focus on the methods used to assess the relationship between device errors and outcomes. Sixteen studies were identified (12 in patients with asthma, one in patients with COPD, and three in both asthma and COPD) with varying study designs, endpoints, and patient populations. Most of the studies reported that inhalation errors were associated with worse disease outcomes in patients with asthma or COPD. Patients who had a reduction in errors over time had improved outcomes. These findings suggest that time invested by healthcare professionals is vital to improving inhalation technique in asthma and COPD patients to improve health outcomes.

## Introduction

Asthma and chronic obstructive pulmonary disease (COPD) are common chronic respiratory diseases that impart an economic and social burden.^1–4^ Rates of asthma vary between countries, affecting 1–18% of the population,^5^ and >10% of adults aged 40 years and over in the general population are estimated to suffer from COPD.^6^

Inhaled medications are the cornerstone of the treatment and management of asthma and COPD.^4,5^ There are many devices for the delivery of inhaled medications, including pressurised metered-dose inhalers (pMDIs), dry-powder inhalers (DPIs), soft-mist inhalers, breath-actuated MDIs, and nebulisers^4,7^; pMDIs and DPIs are the most commonly used.^8,9^ While the wide array of treatments available may be seen as positive, the large number of available devices can result in a certain amount of complexity for prescribers when teaching patients their correct use.

Effective use of inhalers requires patients to follow the prescribed inhalation technique.^7,8^ Errors in device use can result in suboptimal drug delivery, reducing the effective medication dose and thus compromising treatment effectiveness.^9–13^ In this systematic literature review, an error is defined as critical if it has an impact on the effectiveness of the drug. It has been estimated that up to 92% of patients make at least one critical error when using an inhaler, with a higher error rate reported in patients with COPD compared with those with asthma.^9^ The type and frequency of errors vary among devices depending on their characteristics,^11,14–19^ although many common errors are universal, such as failing to exhale before each inhalation and failing to hold the breath following inhalation.^9^ Overall, physicians tend to overestimate good inhalation technique and underestimate errors made by patients.^20^

It is important, therefore, that effective strategies are in place to educate patients on correct inhaler use.^21^ Key guideline groups such as GINA (Global INitiative for Asthma) and GOLD (Global initiative for chronic Obstructive Lung Disease) have made recommendations to monitor inhaler technique in their strategy documents^4,5^; for example, the most recent version of GOLD recommends the assessment and regular evaluation of inhaler technique, with the aim of improving therapeutic outcomes in patients with COPD.^4^ Both GINA and GOLD also recommend that a patient’s ability to use an inhaler device correctly should be integral to decision-making when choosing between available controller medications.^4,5^

Although there is evidence that errors in device use can affect outcomes,^19^ there is no comprehensive overview of the relationship between them. Therefore, this systematic review of the literature was conducted to examine the relationship between device errors and health outcomes (clinical, quality of life [QoL], and economic) in patients with asthma and COPD.

## Literature search

Systematic searches of PubMed, Google, and Google Scholar were conducted in April 2017 (updated December 2017) to identify studies assessing device errors, incorrect handling, or improper technique in patients with asthma or COPD. Additional studies were identified from a recently published systematic literature review of inhaler device errors^9^ and by author recommendation.

PubMed was searched using the search string: (COPD OR chronic obstructive pulmonary disease OR Asthma) AND (error OR mishandling OR erroneous OR incorrect use OR incorrect technique OR improper use OR improper technique OR inadequate technique OR inadequate use OR insufficient use OR insufficient technique OR critical error OR significant error).

Google and Google Scholar were searched using the following key terms, in different combinations: (asthma or COPD device error; device error in asthma or COPD; improper technique in asthma or COPD; inadequate use in asthma or COPD; misuse or mishandling in asthma or COPD; incorrect use in asthma or COPD).

Publications that did not report on clinical trials or clinical studies were excluded from the initial results. Publication titles and abstracts were then screened, and articles that did not link device errors with outcomes of asthma or COPD were excluded. Data were extracted systematically by an independent reviewer using a predefined extraction template (Supplementary Table 1). Odds ratios (ORs) were the principal measures of the relationships between errors and outcomes.

## Identified studies

**Fig. 1:**
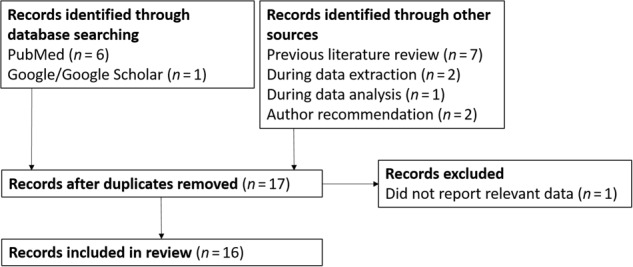
Flow diagram of the literature search

Of the screened publications, a total of 19 relevant articles were identified (Fig. 1). Seven of these were sourced from the database searches (PubMed [*n* = 6], Google/Google Scholar [*n* = 1]), and a further seven were identified from a recent systematic literature review.^9^ The remaining five articles were identified during data extraction (*n* = 2) and data analysis (*n* = 1), and as a recommendation by one of the investigators (MD) (*n* = 2).

Of the 19 relevant articles, three were excluded (duplicates removed [*n* = 2], judged by investigators to not report relevant data [*n* = 1]), leaving a total of 16 unique studies (Fig. 1). These studies reported associations between device errors and clinical outcomes, and were included for discussion in the review. Details of the included studies are shown in Table 1. In general, the studies examined patients with asthma and COPD separately, with most (*n* = 12) studying patients with asthma only (Table 1). Overall, disease severity was not specified in the studies. Most studies included 500 patients or more and focused on adults, with only a few including adolescent patients or patients of all ages (Table 1). In one study that included children below 12 years old, 20% were < 16 years old.^32^ In most studies, patients were recruited from outpatient clinics, mostly in European countries (Table 1). MDIs (alone or with a spacer), breath-actuated MDIs (Autohaler and Easi-Breathe inhaler [trademarks of Teva Pharmaceuticals Ltd]) and DPIs (Diskus [a trademark owned by or licensed to the GSK group of companies], Turbuhaler [trademark of AstraZeneca Ltd], HandiHaler [trademark of Boehringer Ingelheim]) were the most commonly studied inhalers (Table 1).

**Table 1:** Designs of identified studies in asthma and COPD reporting associations between device errors and clinical outcomes

References	Study type	Country	Setting	Patient age	Sample size, *n*	Inhaler(s) studied	Outcomes studied	Device error definition
**Asthma only**								
Giraud & Roche 2002^22^	Clinical cross-sectional	France	OP	> 15 years	4078	pMDI	Clinical, economic	Misusers vs. good users ( ≥ 1 vs. no errors), and poor coordinators vs. good coordinators ( ≥ 1 vs. no errors between actuation and inhalation)
Molimard & Le Gros 2008^23^	Clinical cross-sectional	France	OP	Adults	4362	pMDIs, DPIs	Clinical	Patients making ≥ 1 critical errors vs. patients using inhaler correctly
Giraud, Allaert & Magnan 2011^24^	Clinical cross-sectional	France	OP	Adults	6512	Breath-actuated MDI	Clinical	Patients with suboptimal vs. optimal technique (optimal = correctly following 7-step checklist and avoiding five possible errors)
Natsir et al. 2013^25^	Clinical cross-sectional	Indonesia	OP	Adults	60	NR	Clinical, economic	Patients demonstrating improper inhaler use, evaluated using a checklist based on Global Initiative for Asthma
Al-Jahdali et al. 2013^26^	Clinical cross-sectional	Saudi Arabia	ED	Adults	450	MDIs, DPIs	Clinical, economic	Proper vs. improper inhaler use (proper use = fulfilled all required steps on a device checklist over two trials of using their inhaler)
Baddar, Jayakrishnan & Al-Rawas 2014^27^	Clinical cross-sectional	Oman	OP	12−72 years	218	NR	Clinical	Good inhaler technique (all essential steps performed accurately) vs. poor inhaler technique (any required steps missed/performed inaccurately)
de Tarso Roth Dalcin et al. 2014^28^	Clinical cross-sectional	Brazil	OP	Adults	268	MDIs, DPIs	Clinical	Correct ( < 2 errors) vs. incorrect inhaler technique ( ≥ 2 errors)
Giraud, Allaert & Roche 2011^29^	Prospective clinical (1 month follow-up)	France	Pharmacy	Adults	727	pMDIs, breath-actuated MDIs	Clinical	Optimal use (no errors) vs. non-optimal use ( ≥ 1 critical or non-critical error)
Yildiz et al. 2014^30^	Prospective clinical, longitudinal ( ≥ 6 months follow-up)	Turkey	OP	Adults	572	pMDIs, DPIs	Clinical	Patients making 0–1 basic errors vs. patients making > 1 basic error
Harnett et al. 2014^31^	Prospective clinical, longitudinal (3−4 months follow-up)	Ireland	OP	≥ 16 years	40	pMDIs, DPIs, soft-mist inhaler	Clinical, QoL	Optimal users (no errors) vs. misusers ( ≥ 1 of 10 steps performed incorrectly)
Levy et al. 2013^32^	Retrospective, database (IMPACT), cross-sectional	UK	−	All ages	3981	MDIs, DPIs	Clinical	Patients with correct vs. incorrect technique (incorrect technique = failure of one or more of: inspiratory flow between 10–50 L/min; correct flow for ≥ 1.5 s post-actuation; post-inspiration breath hold for ≥ 5 s)
Price et al. 2017^19^	Retrospective, database (iHARP), cross-sectional	Australia, Europe	−	≥ 16 years	3660	pMDIs and DPIs	Clinical	Frequency of specific errors and device-specific errors
**COPD only**								
Molimard et al. 2017^33^	Clinical cross-sectional	France	OP	> 40 years	2935	pMDIs, Respimat, DPIs	Clinical, economic	Patients with absence of error vs. presence of critical error
**Both asthma and COPD**								
Melani et al. ^2011,8^	Clinical cross-sectional	Italy	OP	> 14 years	1664	MDIs, DPIs	Clinical, economic	Inhaler misuse (patients with presence of error or critical error)
Maricoto et al. 2015^34^	Clinical cross-sectional	Portugal	OP	> 12 years	62	MDIs, DPIs	Clinical	Patients with ≥ 1 error, number of errors committed (0–4)
Roggeri, Micheletto & Roggeri 2016^35^	Clinical cross-sectional	Italy	OP	> 14 years	400	NR	Economic	See above (Melani et al. 2011)^8^

*COPD* chronic obstructive pulmonary disease, *DPI* dry-powder inhaler, *ED* emergency department, *iHARP* Improving Health of At-Risk Rural Patients, *IMPACT* InforMing the PAthway of COPD Treatment, *MDI* metered-dose inhaler, *NR* not reported, *OP* outpatient, *pMDI* pressurised metered-dose inhaler, *QoL* quality of life

## Examination of inhaler errors

Six studies examined critical errors (i.e., the authors specifically stated that ‘critical errors’ were evaluated).^8,19,23,29,33,35^ In the remaining 10 studies, the term ‘critical errors’ was not used;^22,24–28,30–32,34^ however, there was overlap in the actual errors across all studies. Only one study, CritiKal,^19^ identified associations between specific inhaler technique errors and reduced disease control, confirming those errors as critical. Some studies defined errors separately for each device used by patients.^8,19,23,26,28,29^ Errors were generally assessed using checklists for assessment of inhalation technique.^8,19,22–24,26,28,29,31,32,34,35^ Overall, a patient’s technique was judged to be poor (for both critical and non-critical errors) if any one of the required steps was missed or performed inaccurately.^22,23,26–28^ A number of studies in asthma or COPD identified inhaler errors that were established as critical errors in the CritiKal study; these are shown in Table 2.

**Table 2:** Inhaler errors identified as critical in the CritiKal study^19^ that had previously been used in other studies

CritiKal study error	Number of studies that evaluated the error		
	Asthma studies	COPD studies	Both asthma and COPD
Did not remove cap/slide cover open	4^22,24,26,29^	0	1^8^
Insufficient inspiratory effort	5^19,22,26,28,29^	1^33^	1^34^
Did not have head tilted such that chin is slightly upward	2^19,26^	0	0
Did not breathe out to empty lungs before inhalation	5^19,22,24,26,30^	1^33^	1^8^
No breath hold (or holds breath for < 3 s)	7^19,22,24,26,28–30^	1^33^	2^8,34^
Did not seal lips around mouthpiece	5^19,24,26,28,29^	0	1^8^
Incorrect second dose preparation, timing, or inhalation	1^19^	0	0
Exhaled into device before inhalation	2^19,30^	1^33^	0
Dose compromised after preparation because of shaking or tipping (DPIs only)	2^19,28^	0	0
Actuation did not correspond with inhalation, actuation before inhalation (MDI only)	5^19,22,24,26,29^	1^33^	1^8^

*COPD* chronic obstructive pulmonary disease, *DPI* dry-powder inhaler, *MDI* metered-dose inhaler

## Associations between device errors and outcomes

While most studies demonstrated a cross-sectional association between errors and outcomes using several approaches, they did not distinguish between critical and non-critical errors.^22,24–28,30–32,34^ Most commonly, errors were defined dichotomously (i.e., any errors present vs. no errors present), with clinical outcomes compared in patients with and without inhalation errors.^8,19,22–28,32,33^

A less common approach was based on the number of errors (i.e., association between the number of errors and outcomes^22,34^). A few studies focused on specific devices and/or specific errors, and empirically determined the errors that were associated with adverse outcomes.^19,22,32^

Among the longitudinal analyses, two approaches were used. The first approach involved demonstrating an improvement in inhalation technique alongside an improvement in outcomes, without statistically testing the association,^30^ whereas the second approach demonstrated statistical associations between improved inhalation technique and improved outcomes over a period of 1 month or more.^29,31^ Prospective studies generally involved participant training, and before/after comparisons of inhalation technique and outcomes.^29–31^ However, two longitudinal studies examined the evolution in error rate over extended periods of time (3−4 and ≥ 6 months, respectively^30,31^).

## Effect of device errors on outcome measures

Most of the studies assessed the effects of device errors on the clinical outcome of disease control in asthma and COPD (Table 1). Measures of asthma control were the Asthma Control Test (ACT; *n* = 6),^8,26,27,30,31,34^ the Asthma Control Questionnaire (ACQ; *n* = 3),^24,29,31^ study-specific questionnaires (*n* = 3),^19,22,23^ GINA category (*n* = 2),^28,32^ Control of Allergic Rhinitis and Asthma Test (*n* = 1),^34^ and any unscheduled medical intervention due to respiratory disease (*n* = 2).^8,19^ COPD control was assessed using the modified Medical Research Council (mMRC) Dyspnoea Scale (*n* = 2)^8,34^ and the number of COPD exacerbations (*n* = 1).^33^ Only one study evaluated the clinical outcome of QoL in patients with asthma, reporting the association between device errors and QoL using the Asthma Quality of Life Questionnaire (AQLQ)^31^ (Table 1). Six studies assessed healthcare resource utilisation (HRU) as the clinical outcome in asthma and/or COPD (Table 1). Economic outcome measures were emergency room (ER) visits (*n* = 6),^8,22,25,26,33,35^ hospitalisations (*n* = 2),^8,35^ and prescriptions (antimicrobial agents, steroids, *n* = 2)^8,35^; one study examined costs associated with increased HRU due to critical errors in both asthma and COPD patients.^35^

Key findings of studies in asthma and COPD are shown in Supplementary Tables 2 and 3. Data supporting an association between inhalation errors and worse disease outcomes were reported in almost all studies, and patients in the longitudinal studies who had a reduction in errors over time had improved outcomes, irrespective of endpoint. Despite differences in study designs across the evaluated studies, the magnitude of effect appeared to be similar across the different endpoints. For example, reported ORs of the relationship between device errors and worse outcomes typically ranged from 1.46 to 1.73 for ACT score,^8^ and 1.30 to 1.56 for study-specific questionnaires assessing asthma control.^19^ ORs linking critical inhaler errors to mMRC Dyspnoea score^8^ or severe COPD exacerbations (requiring hospitalisation/ER visit)^33^ were 1.10 and 1.86, respectively. Two studies failed to report an association between errors and outcomes (one in asthma patients,^31^ and another in the COPD cohort of a mixed asthma/COPD study^34^); however, both had small sample sizes (40 and 27 [COPD] patients, respectively), and were possibly underpowered.^31,34^

Inhaler technique, asthma outcomes and treatment adherence have all been linked to one another^34^; however, only five of the evaluated studies measured treatment adherence, all of which measured patient-reported adherence.^19,23,27,29,33^ Giraud et al.^29^ found that training patients in inhaler technique led to statistically significant improvements in ACQ score, patient-reported adherence, and the number of patients demonstrating optimal technique (all *p* < 0.001).

## Discussion

The majority of studies identified from the literature reported an association between inhalation errors and worse disease outcomes in patients with asthma or COPD, and the magnitude of effect was similar across the different endpoints studied. Most of the studies were cross-sectional and were conducted in pulmonology clinics/outpatient departments. A few prospective, longitudinal studies, and some database analyses, were also identified. There are advantages and disadvantages to each study design that should be considered when interpreting the literature. For example, it is difficult to infer causality using a cross-sectional approach, due to lack of temporality. This approach can also be subject to recall bias (especially for HRU studies). In general, outcome data (e.g., number of hospitalisations, ER visits etc.) in the identified studies relied upon retrospective recollection by patients, and were often limited to events in the recent past (1−3 months). Furthermore, while database analyses provide readily available data, few databases capture data on device errors, and as a result, associated information on inhalation technique. Prospective studies can overcome the issue of temporality by providing more reliable data on outcomes, especially those related to HRU. However, they are operationally more challenging to conduct, and do not provide results as quickly as cross-sectional or database analyses. Longitudinal studies, on the other hand, can evaluate the long-term impact of errors and causality, and are able to address a variety of research questions. For example, longitudinal studies can be used to measure the effects of interventions that target inhaler errors.

Despite guidance by GINA, GOLD, and national asthma and COPD guidelines, inhaler errors made by patients are still prevalent, and the type and incidence of errors has not changed considerably over the past 40 years.^36^ As little has improved over the last decade,^20^ further efforts to support patients in using their inhaler(s) or an alternative approach to delivering inhaled medications is needed to resolve this ongoing issue.^13^ For example, investing in training time for healthcare professionals could help to enhance patient support and improve overall inhalation technique. Efforts have also been made to develop simplified regimens with fewer inhalers,^13^ and to provide inhalers that are more intuitive to use.^37^ Both strategies have the potential to reduce the number of errors made by patients, subsequently enhancing the effectiveness of the medication and associated patient outcomes.

Alternative approaches to help patients retain the skills needed to use their device correctly should also be considered. The recent development of electronic devices that attach to existing inhalers, or the development of integrated inhalers that measure inhalation characteristics, can provide direct feedback to patients on their inhaler technique. This has the potential to reduce errors, as previous studies have shown that regular feedback can improve the retention of correct inhaler technique.^13^

As treatment adherence has an impact on asthma outcomes,^34^ and an association has been demonstrated between adherence and inhaler technique (poor adherence was linked with poor technique, and good adherence with good technique),^29^ it is important to consider the potential for treatment adherence to amplify associations between device errors and asthma outcomes.

There is a lack of consistency in the literature regarding which errors are defined as critical. Errors should be assessed using a single checklist that is standardised as much as possible across the different devices. One unified definition for ‘critical errors’ should be used; for example, the CritiKal study catalogued errors that were related to poor asthma control.^19^ Studies should report errors individually for the various devices in order to identify persistent problems and better target device training. A recent systematic review and meta-analysis estimating error rates in MDIs and DPIs failed to show a difference between various inhalers due to a relatively limited body of evidence. However, it did confirm that overall and critical error rates were unacceptably high across all devices.^9^ Most of the errors in the CritiKal study were generic to the type of inhaler (DPI or MDI) rather than device specific.^19^

A limitation of this systematic review was that a meta-analysis of the literature could not be performed due to heterogeneity in study designs, outcomes, and patient populations studied, although these may become viable in the future as study designs become more uniform.

Published data from studies in asthma and COPD demonstrate the presence of an association between inhalation errors and outcomes, with an apparent relationship between a reduction in errors and improvement in outcomes, irrespective of endpoint. The magnitude of effect is similar across the different endpoints. Therefore, investment of time by healthcare professionals is vital to improving inhalation technique in asthma and COPD patients in order to improve health outcomes. The methodology used to study the relationship between inhaler errors and outcomes varies widely. Therefore, there is a need for greater standardisation of the methods used to assess inhaler errors across the spectrum of devices and outcome measures. Future research should also focus on identifying ways to improve device handling.

## Electronic supplementary material

Supplementary Table 1

Supplementary Tables 2&3

## Data Availability

All publications identified in the systematic literature review are accessible via the public databases (PubMed, Google/Google Scholar), or via the articles’ respective journal websites. All of the included data were sourced from these publicly available articles.

## Electronic supplementary material

**Supplementary information** accompanies the paper on the *npj Primary Care Respiratory Medicine* website (10.1038/s41533-018-0110-x).
