# Supplementary material for: Systematic review of association between critical errors in inhalation and health outcomes in asthma and COPD
Source: NPJ Prim Care Respir Med. 2018 Nov 16;28:43. doi: 10.1038/s41533-018-0110-x (PMC6240098; doi:10.1038/s41533-018-0110-x)
Supplement: Supplementary file 2 — Supplementary Tables 2&3 [file 41533_2018_110_MOESM2_ESM.docx]

**Supplementary Table 2.** Key findings of studies including patients with asthma

| **Ref** | **Patients, *n*** | **Key conclusions** | | |
| --- | --- | --- | --- | --- |
|  |  | **Clinical outcomes** | **QoL** | **HRU outcomes** |
| **Clinical cross-sectional studies** | | | | |
| Giraud & Roche 2002^22^ | 4078 | - Asthma was less stable in pMDI misusers (patients with ≥1 error) than in good users (AIS 3.93 vs. 2.86; *p<*0.001). Among misusers, asthma was less stable in poor coordinators (AIS 4.38 vs. 3.56 in good coordinators; *p<*0.001) - There were significant differences in AIS according to the presence or absence of each single error or omission in inhalation technique (*p=*0.03 for cap removal and *p<*0.00001 for all others) - Linear regression analysis showed that the number of errors in inhalation technique correlated to the AIS (*r=*0.3; *p<*0.0001) | NR | - In misusers as compared with good users, more frequent β_2_-agonist use, recent worsening of asthma and occurrence of serious exacerbations (all *p<*0.01) were reported - ER visits were more frequent in misusers with poor coordination (≥1 error between actuation and inhalation) than in misusers without poor coordination or good users (*p<*0.00001) |
| Molimard & Le Gros 2008^23^ | 4362 | - >20% of patients were using their inhaler device incorrectly (Turbuhaler 37.1%; MDI 28.1%; Diskus 21.3%; Aerolizer 7.4%) and this was associated with a 0.84-point increase in ACS - Mean ACS was 2.95 (95% CI 2.88, 3.02) and asthma control was unsatisfactory (i.e. ACS ≥4) in 41.1% of patients, with higher proportions in smokers, non-compliant patients, and those misusing their device - Asthma control (study-specific questionnaire) was inadequate in 53.3% of patients committing a critical error, compared with 37.3% of those who were using their inhaler correctly | NR | NR |
| Giraud, Allaert & Magnan 2011^24^ | 6512 | - The proportions of patients with suboptimal technique rose as the ACQ score rose from 732/1512 (48.4%) of patients with ACQ score of 0−1 to 174/265 (65.7%) of patients with ACQ score ≥4 | NR | NR |
| Melani *et a*l. 2011^8^ | 703 | - There was an association between ACT score and risk of critical error both overall (asthma, COPD, and ‘other’ patients [n=1664], OR 1.53 ± 0.14; *p<*0.0001) and in patients with a primary diagnosis of asthma (OR 1.73 ± 0.26; *p<*0.0001) | NR | - Critical errors in inhalation technique were significantly associated with increased risk of hospitalisation (*p*=0.001), ER visits (*p*<0.001), use of antibiotics (*p*<0.001) and courses of oral corticosteroids (*p*<0.001) |
| Natsir *et al.* 2013^25^ | 60 | - Improper use of asthma inhaler devices was observed in 36 (60%) patients and was associated with level of formal education (*p=*0.016), irregular clinic follow-ups (*p=*0.01) and uncontrolled asthma (ACT score <15; *p=*0.04) | NR | - Improper use of asthma inhaler devices was associated with ER visits (*p=*0.00) |
| Al-Jahdali *et al*. 2013^26^ | 450 | - Improper use of asthma inhaler devices was observed in 45% of the patients and was associated with irregular clinic follow-ups (*p=*0.0001), lack of asthma education (*p=*0.0009), uncontrolled asthma (ACT score ≤15) (*p=*0.001) and duration of asthma of <52 weeks (*p=*0.005) | NR | - Improper use of asthma inhaler devices was associated with ≥3 ER visits (*p=*0.0497). |
| Baddar *et al*. 2014^27^ | 218 | - Patients with poor inhaler techniques demonstrated poor control according to ACT score (OR 5.3; 95% CI 2.05–14.8; *p<*0.001) - In patients with good asthma control, 38% (35) displayed good inhaler technique as well as compliance and 41.3% (38) had a good technique and partial compliance | NR | NR |
| de Tarso Roth Dalcin *et al*. 2014^28^ | 268 | - Incorrect inhaler technique was associated with poor GINA-derived asthma control. The proportion of patients with uncontrolled asthma was significantly higher among those with incorrect inhaler technique (*p=*0.007) - FVC, FEV_1_ and FVC/FEV_1_ were lower in patients with correct versus incorrect technique, but there was no significance difference | NR | NR |
| Maricoto *et a*l. 2015^34^ | 35 | - There was an association between lower number of errors and better clinical control, either by the ACT becoming significant from 3 errors committed with a mean difference of 7.5 (± 3.5) points (*p=*0.032, ANOVA), or by CARAT becoming significant at 4 errors with an average difference of 8.5 (± 3) points (*p=*0.008, ANOVA) | NR | NR |
| Roggeri, Micheletto & Roggeri 2016^35^ | 200 | NR | NR | - Among asthma patients with (*n*=100) vs without (*n*=100) ≥1 critical errors, critical errors were associated with an excess of hospitalisation (19 more), ER visits (26.5 more), antimicrobial courses (4.5 more) and courses of corticosteroids (21.5 more) - These differences in resource consumption were associated with a yearly incremental healthcare cost for 100 patients, due to inhalation errors, of €44,104 |
| **Prospective clinical studies** | | | | |
| Giraud, Allaert & Roche 2011^29^ | 727 | - At 1 month, mean (SD) ACQ score had improved from a baseline score of 1.8 (1.2) to 1.4 (1.1) (*p<*0.001). Importantly, greater change was observed in patients with improved inhaler technique vs. those without. Similar results were observed for Morisky score | NR | NR |
| Yildiz *et al.* 2014^30^ | 572 | - ACT asthma control (overall 61.5% at baseline, and increased to 87.3% during follow-up) was better, with significant improvement in technique and decrease in basic errors to the range 0–1, regardless of inhaler type | NR | NR |
| Harnett et al. 2014^31^ | 40 | - At the initial assessment, 63% of the patients were classified as incorrectly using their inhaler which decreased to 20% at follow-up, indicating an overall significant improvement in inhaler usage post-training (*p=*0.003). ACQ scores improved significantly from median [IQR] 2.70 [1.66] to 2.00 [1.90] (*p=*0.002) | - Correlation between improved AQLQ scores and improved inhaler technique at follow-up did not reach statistical significance (*r=*0.37; *p=*0.3) - Patients’ AQLQ score strongly correlated with ACQ score (r=0.8), with poorer AQLQ coinciding with decreasing asthma control (*p<*0.001) | NR |
| **Database studies** | | | | |
| Levy *et al.* 2013^32^ | 3981 | - Incorrect inhaler use was found in 4× as many patients with uncontrolled asthma according to GINA criteria and >2× as many patients with partly controlled asthma (*p<*0.0001) - Significantly more patients who had ≥1 asthma exacerbation (68%) failed their first inhaler technique test (*p=*0.03) - 67% of those prescribed a short course of prednisolone in the 3 months prior (OR 0.50–0.89; *p<*0.05) failed their first AIM inhaler technique test when attending the clinic - Patients using spacers with their pMDIs had better control of their asthma than those not using spacers (68% vs. 51%; *p*<0.0001) | NR | NR |
| Price *et al.* 2017^19^ | 3660 | - Insufficient inspiratory effort was common (32−38% of DPI users) and was associated with uncontrolled asthma (study-specific questionnaire) with Turbuhaler (adjusted OR 1.30; 95% CI 1.08–1.57) and Diskus devices (adjusted OR 1.56; 95% CI 1.17, 2.07) and increased exacerbation rate - In MDI users, actuation before inhalation (24.9% of patients) was associated with uncontrolled asthma (OR 1.55; 95 CI 1.11–2.16) - Several more generic and device-specific errors were also identified as critical | NR | NR |
| ACQ, Asthma Control Questionnaire; ACS, Asthma Control Score; ACT, Asthma Control Test; AIM, Aerosol Inhalation Monitor; AIS, Asthma Instability Score; ANOVA, analysis of variance; AQLQ, Asthma Quality of Life Questionnaire; CARAT, Control of Allergic Rhinitis and Asthma Test; CI, confidence interval; COPD, chronic obstructive pulmonary disease; DPI, dry powder inhaler; ER, emergency room; FEV_1_, Forced expiratory volume in 1 s; FVC, forced vital capacity; GINA, Global Initiative for Asthma; HRU, healthcare resource utilisation; IQR, interquartile range; MDI, metered-dose inhaler; NR, not reported; OR, odds ratio; pMDI, pressurised metered-dose inhaler; QoL, quality of life; RR, rate ratio; SD, standard deviation | | | | |

**Supplementary table 3.** Key findings of studies including patients with COPD

| **Ref** | **Patients, *n*** | **Key conclusions** | |
| --- | --- | --- | --- |
|  |  | **Clinical outcomes** | **HRU outcomes** |
| **Clinical cross-sectional studies** | | | |
| Melani *et a*l. 2011^8^ | 864 | - An association between ACT score and risk of critical error in patients with COPD (OR 1.46 ± 0.18; *p<*0.005) - No association between COPD patients with critical inhaler errors and mMRC dyspnoea score (OR 1.10 ± 0.07) | - Inhaler misuse was associated with increased risk of hospitalisation (*p<*0.001), ER visits (*p<*0.001), courses of oral steroids (*p<*0.001) and antimicrobials (*p<*0.001) in the overall (asthma, COPD and ‘other’) population |
| Maricoto *et al.* 2015^34^ | 27 | - No significant relationship between clinical control and quality of inhalation technique - CAT and mMRC scores associated with different numbers of errors, which did not show a significant difference | NR |
| Roggeri, Micheletto & Roggeri 2016^35^ | 200 | NR | - Among patients with (*n*=100) vs without (*n*=100) ≥1 critical error, critical errors were associated with an excess of hospitalisations (11.5 more), ER visits (13 more), antimicrobial courses (19.5 more) and corticosteroids courses (47 more) - These differences in resource consumption were associated with a yearly incremental healthcare costs for 100 patients, due to inhalation errors, of €23,444 |
| Molimard *et al.* 2017^33^ | 2935 | - Handling errors of inhaler devices are underestimated in real life and are associated with an increased rate of severe COPD exacerbation | - Proportion of patients requiring hospitalisation or ER visits in the past 3 months for severe COPD exacerbation was 3.3% (95% CI 2.0–4.5) in the absence of error and 6.9% (95% CI 5.3–8.5) in the presence of critical error (OR 1.86; 95% CI 1.14–3.04; *p<*0.05) |
| ACT, Asthma Control Test; CAT, COPD Assessment Test; CI, confidence interval; COPD, chronic obstructive pulmonary disease; ER, emergency room; HRU, healthcare resource use; mMRC, modified Medical Research Council; NR, not reported; OR, odds ratio | | | |
